# 3D visual cueing shortens the double support phase of the gait cycle in patients with advanced Parkinson's disease treated with DBS of the STN

**DOI:** 10.1371/journal.pone.0244676

**Published:** 2020-12-31

**Authors:** Kamila Poláková, Evžen Růžička, Robert Jech, David Kemlink, Jan Rusz, Eva Miletínová, Hana Brožová

**Affiliations:** 1 Department of Neurology and Center of Clinical Neuroscience, 1st Faculty of Medicine and General University Hospital, Charles University, Prague, Czech Republic; 2 Department of Circuit Theory, Faculty of Electrical Engineering, Czech Technical University in Prague, Prague, Czech Republic; São Paulo State University (UNESP), BRAZIL

## Abstract

**Background:**

Gait disturbances have emerged as some of the main therapeutic concerns in late-stage Parkinson’s disease (PD) treated with dopaminergic therapy and deep brain stimulation (DBS). External cues may help to overcome freezing of gait (FOG) and improve some of the gait parameters.

**Aim:**

To evaluate the effect of 3D visual cues and STN-DBS on gait in PD group.

**Methods:**

We enrolled 35 PD patients treated with DBS of nucleus subthalamicus (STN-DBS). Twenty-five patients (5 females; mean age 58.9 ±6.3) and 25 sex- and age-matched controls completed the gait examination. The gait in 10 patients deteriorated in OFF state. The severity of PD was evaluated using the Unified Parkinson's Disease Rating Scale (UPDRS) and Hoehn and Yahr (HY). The PD group filled the Falls Efficacy Scale-International (FES) and Freezing of Gait Questionnaire (FOGQ). Gait was examined using the GaitRite Analysis System, placed in the middle of the 10m marked path. The PD group was tested without dopaminergic medication with and without visual cueing together with the DBS switched ON and OFF. The setting of DBS was double-blind and performed in random order.

**Results:**

The UPDRS was 21.9 ±9.5 in DBS ON state and 41.3 ±13.7 in DBS OFF state. HY was 2.5 ±0.6, FES 12.4 ±4.1 and FOGQ 9.4 ±5.7. In the DBS OFF state, PD group walked more slowly with shorter steps, had greater step length variability and longer duration of the double support phase compared to healthy controls. The walking speed and step length increased in the DBS ON state. The double support phase was reduced with 3D visual cueing and DBS; the combination of both cueing and DBS was even more effective.

**Conclusion:**

Cueing with 3D visual stimuli shortens the double support phase in PD patients treated with DBS-STN. The DBS is more effective in prolonging step length and increasing gait speed. We conclude that 3D visual cueing can improve walking in patients with DBS.

## 1. Introduction

People with Parkinson’s disease (PD) in advanced stages suffer frequently from gait disturbances, which could be partly influenced by dopaminergic medication. Gait impairment in PD is characterized by hypokinesia with lower speed, shortened step length, narrow step width, reduced counter-rotation of the trunk and decreased arm swing [1]. Some people with PD may also develop freezing of gait (FOG), that is short, unpredictable periods where they are unable to voluntarily initiate or maintain gait [2]. FOG mainly occurs when the patient is not on dopaminergic medication, and can be provoked by specific triggers including a specific motor action (e.g., turning), other factors that could be cognitive (e.g., dual-tasking), affective (e.g., threatening situations) and environmental (e.g., narrow doorways) [2, 3].

Pathophysiology of gait impairment in PD is complex and involves several cerebral structures across the locomotor network. A deficiency of the internal cueing mechanism due to basal ganglia disorder is suggested to lead to inappropriate scale and timing of the automatic movement sequences [1]. With the loss of dopaminergic neurons, some gait parameters (speed, stride length) [4] respond to dopaminergic medication, whereas temporal parameters (cadence, step and swing duration, double support time) improve to a lesser extent [5] or not at all [6].

Treatment with invasive methods, such as deep brain stimulation (DBS) of the subthalamic nucleus (STN-DBS) in PD, is effective in alleviating disabling motor complications [7, 8]. The treatment may improve posture, quiet standing postural control and the parameters of gait that were responsive to levodopa prior to surgery [9, 10]. STN-DBS can also reduce FOG in the OFF-medication state [7, 11–13]. The effects of DBS on postural stability and gait, however, tend to decrease with time [14–16] and the long-term effect of STN-DBS on FOG in the ON medication state is disputable [17]. A postoperative deterioration of gait was documented in a considerable number of PD patients after implantation of STN-DBS [8, 11, 14, 18, 19] with higher risk of falls, worsening of dynamic postural control [8, 11, 14, 18–20] and even new development of FOG [7, 11, 21].

To overcome FOG, some PD patients use various strategies, involving particular internal or external cues, which help to initiate or maintain gait. These include, for example, stepping over obstacles, modification of gait pattern or use of alternative methods of locomotion, i.e. cycling or skating [22–24]. The external cues are usually less attentionally demanding. The precise mechanisms underlying the effectiveness of cueing is unclear, however there are some reasonable explanations, e.g. involvement of goal-directed behavior with circumnavigation of parts of the basal ganglia, mechanisms assisting in filtering information [25] or optic flow compensating sensory deficit [26, 27].

The immediate effect of external cues on walking speed, step length, and step frequency of the gait of PD patients has been documented [28–30]. A variety of wearable devices were lately presented, with smaller [31] or greater effect on FOG [32, 33]. Case reports suggested that 3D visual cues might be more effective in reducing FOG than 2D cues [22, 23]; however detailed studies are not available.

We aimed to evaluate the effect of 3D visual cues and STN-DBS on gait in patients with advanced PD. We hypothesized that the 3D visual cueing would mainly reduce the frequency and severity of FOG, while STN-DBS would more likely influence the dopa-sensitive gait parameters (i.e. speed, step length). The mechanisms of both methods are different, and the benefit could be thus complementary. Both methods are well-established, nevertheless a more detailed analysis of the exact effect of visual cueing on gait parameters in the native state (DBS OFF) compared to the changes caused by DBS has not yet been documented.

## 2. Materials and methods

### 2.1. Participants

We investigated 35 non-demented PD patients (6 females; mean age 60.6 ±6.3, disease duration 17.8 ±4.5 years), treated with dopaminergic medication together with STN-DBS. The exclusion criteria were dementia and any other neurological or non-neurological condition that may affect gait. The severity of PD was evaluated using the Unified Parkinson's Disease Rating Scale (UPDRS) and Hoehn and Yahr (HY) stage. Subjects filled in a shortened version of the Falls Efficacy Scale-International (FES-I, score range 7–28) and the Freezing of Gait Questionnaire (FOGQ). The final PD group included 25 subjects (5 females; mean age 58.9 ±6.3) who completed the whole study protocol. Ten recruited PD patients did not complete the gait examination, as they were unable to walk independently when the DBS was switched OFF. The details of the PD group can be found in Table 1 and S1 Table.

**Table 1:** Characteristics of the PD group.

	Complete evaluation (n = 25)		Unable to complete (n = 10)	
	Mean ±SD	(min-max)	Mean ±SD	(min-max)
**Age (years)**	58.9 ±6.3	(50–73)	65.0 ±4.1	(58–72)
**Disease duration (years)**	18.0 ±4.9	(10–31)	17.5 ±3.8	(12–24)
**DBS duration (years)**	4.8 ±3.7	(0.7–15)	14 ±3,9	(10–23)
**L-Dopa Eq (mg)**	995.2 ±402.2	(399–1896)	1500 ±829,8	(399–3171)
**TEED (mA2.Ω.Hz.ms)**	100.3 ±63.7	(13.3–222.1)	112 ±48,8	(40.0–196.0)
**UPDRS I** (range 0–16)	1.3 ±1.5	(0–5)	1.3 ±1.0	(0–3)
**UPDRS II** (range 0–52)	10.0 ±5.6	(1–21)	17.8 ±4.9	(12–31)
**UPDRS III ON** (range 0–56)	21.9 ±9.5	(7–43)	32.2 ±12.3	(18–56)
**UPDRS III OFF** (range 0–56)	41.3 ±13.7	(12–66)	62.7 ±7.8	(54–79)
**Hoehn and Yahr** (range 1–5)	2.5 ±0.6	(1.5–4)	3.2 ±0.6	(2.5–4)
**FOGQ** (range 0–24)	9.4 ±5.7	(1–24)	15.0 ±2.3	(12–20)
**FES** (range7-28)	12.4 ±4.1	(7–22)	17.1 ±6.5	(8–28)

DBS, deep brain stimulation; L-Dopa Eq, levodopa equivalent; TEED, total electrical energy delivered; UPDRS, The Unified Parkinson's Disease Rating Scale; MMSE, Mini-Mental State Examination; FOGQ, Freezing of Gait Questionnaire; FES, Falls-Efficacy Scale. Complete evaluation, 25 PD patients who finished the investigation. Unable to complete, 10 PD patients who were not able to complete the investigation due to deterioration of gait in DBS OFF state.

Twenty-five age- and sex-matched healthy controls (5 females; mean aged 59.1±6.2 years) were involved for evaluation of the gait.

The study was approved by the Ethics Committee of the General University Hospital in Prague (86/16 VES 2017 AZV-VFN) and was in compliance with the Declaration of Helsinki. Written informed consent was obtained from all participants.

### 2.2. Assessment and experimental protocol

Gait was tested using the GaitRite Analysis System, a 4.9 m long pressure-sensitive walking mat was placed in the middle of the 10 m marked path. PD group and healthy controls were instructed to wear comfortable shoes and walk over the mat at self-preferred comfortable speed.

For visual cueing of gait, 16 squared wooden rods sized 2x2x100 cm were placed at distances of 60 cm [34] across the walking pathway, perpendicular to the walking direction. The scheme of the investigated path is presented on the S1 Fig.

The examination of gait in the PD group was performed in the medication-OFF state following a withdrawal of dopamine agonist for 72 hours, and the last dose of levodopa was taken 12 hours before the testing. The examination in the PD group was held in four conditions: (i) with STN-DBS switched off (DBS OFF) without cueing, (ii) DBS OFF with visual cueing of gait, (iii) STN-DBS switched on (DBS ON) without cueing, (iv) DBS ON with cueing. The cued gait trial in each condition always followed after gait without cueing. The setting of DBS was double-blind and performed in random order. Gait examinations were carried out at least 90 minutes after each change of DBS setting, when the patients were asked to relax. Gait tests in each condition were repeated twice. DBS ON was set individually to optimal parameters at standard pulse frequency (130Hz), with the voltage in the range of 1.0 to 4.1V or current 1.2 to 3.0mA, pulse width 60, 90 or 120μs.

In healthy controls, gait was tested in two conditions only, without and with visual cues.

### 2.3. Data analyses

Computerized analysis of gait recordings was performed using the GaitRite software. We gathered spatial (step length and variability; base width) and temporal characteristics (step time and variability, double support time, gait speed and cadence) of gait.

### 2.4. Statistical analysis

Descriptive statistics for the collected data was performed to determine the distribution of demographic, clinical and gait variables. Since ten of the recruited patients were unable to complete the gait examination in the DBS OFF state, we did not include their data in the final analysis. Gait parameters were calculated as an arithmetic mean value of results in two trials in each condition. Given the number of individuals tested and the normal distribution of residuals in the models used, the application of the parametric tests is justified. To exclude the effect of the different height we used repeated measures analysis of covariance (ANCOVA) tests factored for disease status corrected for leg length to compare difference in the gait parameters in the PD and control group, for evaluation of individual differences, p-value of Tukey HSD test was reported. Paired T-tests were performed for some parameters in the PD group. Bonferroni correction for multiple comparisons was performed for 8 independent tests. STATISTICA data analysis software system, version 12.0. (statsoft.com) was used for statistical analysis of data.

## 3. Results

At baseline, the majority of the PD group (23 of 25) mentioned the fear of falling, according to the FES. Twenty PD patients admitted FOG occurrence at least once per month according to FOGQ. Even so, only 3 of them developed FOG on the investigated path during a comfortable walk in the DBS OFF state; the effect of 3D visual cueing and DBS was thus insignificant.

In the PD group, the gait in the DBS OFF condition was significantly slower (p = 0.00044, F (1,47) = 14.276, η^2^ = 0.23) with shorter steps (p = 0.000002, F (1,47) = 28.86, η^2^ = 0.380), greater step length variability (p = 0.002, F (1,47) = 10.61, η^2^ = 0.184), and longer duration of the double support phase (p = 0.025, F (1,47) = 5.36, η^2^ = 0.102) compared to healthy controls. No difference was found in cadence, step time or base of support.

The speed of gait (p = 0.000168, F (1,23) = 3.49, η^2^ = 0.132) and step length (p = 0.000164, F (1,23) = 3.90, η^2^ = 0.145) significantly increased in the DBS ON versus DBS OFF condition, however both parameters remained mostly unchanged with 3D visual cueing.

**Fig 1:**
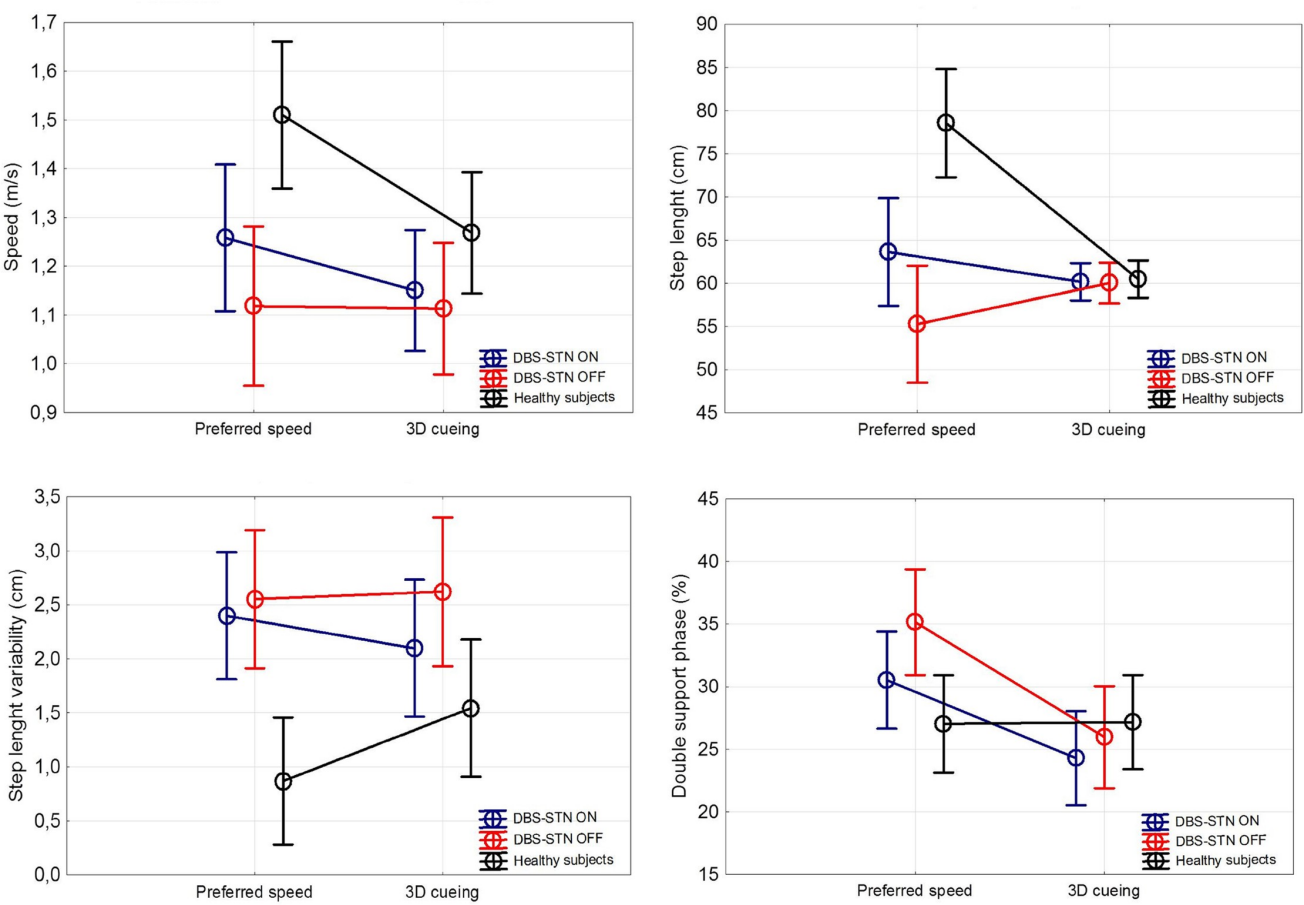
The comparison of effects of 3D visual cueing and DBS on gait. ANCOVA factored for disease status corrected for leg length. DBS OFF, PD group with deep brain stimulation turned off; DBS ON, PD group with deep brain stimulation turned on; Preferred speed, gait without cueing; 3D cueing, gait with presence of 3D visual cues.

The double support phase (F (1,23) = 1.02, η^2^ = 0.042) was significantly reduced during gait with 3D visual cueing (p = 0. 000164, Tukey HSD test) as well as in DBS ON state (p = 0.000165, Tukey HSD test) compared to DBS OFF in the PD group; however the difference in double support phase reduction was greater with visual cueing than in DBS ON (p = 0.0095, t (24) = 2.8). Step length variability was not significantly affected in the PD group by either 3D cueing or DBS. For gait parameters see Fig 1 and S1 Table.

Ten patients, out of those recruited, were unable to complete the gait examination in the OFF state due to inability to walk independently. Those patients were significantly older, with longer duration of PD and higher severity of the disease. They also had a higher score in FES as well as FOGQ; for detailed characteristics see Table 1.

## 4. Discussion

Gait disturbances have emerged as some of the main therapeutic concerns in late-stage PD even when treated with DBS, because they impair mobility, lead to falls and have a strong impact on quality of life [10]. Subjective gait or balance difficulties including a fear of falling were also reported by the majority of the PD group in the present study. The gait analysis revealed slower gait with shortened step length, longer duration of double support phase and higher step length variability in the PD group compared to healthy controls in accordance with earlier studies [35–37]. Contrary to our hypothesis, we did not prove a significant influence of either DBS or visual cueing on the FOG, as the number of captured episodes was too low. The majority of the PD group admitted FOG in the daily life at baseline, but only a very few developed FOG on the investigated path. We did not involve specific triggers, i.e. turns or narrow passage. Moreover, a higher attention during the investigation in laboratory condition may also played a role. Even the inability of the 10 most affected PD patients to complete the examination provides further explanation.

The main effect of the 3D cueing in the PD group was the significant reduction of prolonged double support. Double support phase represents the period of gait cycle when both feet are in contact with the ground and provide more control over the center of the mass movement. During this phase, the patients may correct present disturbances [38]. Its longer duration is thus attributed to impairment of dynamic balance [39] and postural control [40]. With decreased walking speed, the double support phase may also increase, both in time and as a percentage of the gait cycle [38]. However, the speed of the PD group remained unchanged during the 3D visual cueing in the present study in contrast to DBS. Therefore, we assume that the 3D visual cueing mainly improved the stability of gait associated with the shortening of the double support phase. The effect of DBS on the double support phase was also present; however, the visual cueing proved to have greater effect on this parameter.

The external cues also showed improvement in speed, step length, and cadence in other studies [28–30]. The step length was, however, not affected by the external 3D visual cues in this study. We used fixed distance of the cues, which was only slightly longer than the average step length of the PD group in DBS OFF state. The DBS stimulation prolonged the step length significantly and increased walking speed, as expected as both parameters (step length and speed) are highly dopa-responsive [4–6]. The effect on different gait parameters is consistent with different mechanisms of both methods. STN-DBS activates neurons in the basal ganglia-thalamic-cortical system [41] and thus modulates cortical areas that participate in the preparation and execution of movements. Besides, STN-DBS modifies pallido-nigrofugal projections to brainstem areas, which participate in locomotor pattern generation [42]. Visual cues on the other hand work as external drivers that facilitate a compensatory shift to goal-directed control of movement during gait. Focused attention to the stepping process enables the shift from the typical automatic control of gait into a more conscious movement [1]. The striatal dopamine depletion occurring in PD is supposed to impair internal drivers that regulate the automaticity of gait. More conscious motor control strategy during cued gait may help to bypass the impaired basal ganglia. This theory is supported by increased cortical activity observed during targeted movement [43, 44]. Other theory suggests that augmented visual feedback of selfmotion compensates for weakened proprioceptive signal from the lower limbs in PD due to the sensory deficit [26]. Optic flow information received by the participant was revealed to be an important aspect contributing to the improvement of gait with visual cues as they use the visual information from visual cues in both on-line and feedforward fashions [27].

Last documented altered parameter in our PD group was greater step length variability, which was not affected either by 3D cueing or DBS. Increased gait variability is described throughout the disease course and its magnitude tends to increase with disease severity [45]. It is suggested to be an important predictor of the risk of falling [45, 46] and is considered as an expression of reduced automatic control of walking. Unlike other spatiotemporal features, variability is relatively independent of stride length. Lately, there is a growing evidence, that the traditional linear measurement can mask the true structure of motor variability since the biomechanical data from a few numbers of strides are averaged during the analysis. Recent research of gait dynamics based on nonlinear methods revealed that fluctuations in certain gait parameters are not random but display a deterministic behavior, which may degrade in some condition resulting in local instability [45, 47]. Those methods are, however, based on a larger number of gait cycles.

One of the main limitations of the study was, that we were unable to investigate the 10 most affected patients due to the deteriorated motor status when their therapy (medication, DBS) was discontinued. The worsened motor symptoms in the OFF state in the rest of the PD group allowed us to perform only 2 trials in each condition with limited number of gait cycle.

Although we have not been able to directly verify the effects on freezing, we conclude that 3D visual cueing can aid walking in PD patients treated with DBS. Combination of the two methods is beneficial as DBS improves mainly the dopa-sensitive parameters (gait speed, step length) and visual cueing additionally improves the stability associated with normalization of the double support phase. Nevertheless, further research in a larger group is recommend. Essential is also to focus on optimal distance of visual cueing, including dynamic adjustment and performance of the nonlinear measurements of gait for deeper understanding of variability. A long-term effect of visual cues on the gait if used during rehabilitation is expected, however deeper evaluation is needed.

## 5. Conclusion

Cueing with 3D visual stimuli shortened the double support phase of the gait cycle in PD patients treated with STN-DBS. The DBS was more effective in prolonging step length and increasing gait speed. Combination of the two methods is beneficial, however further research of optimal distance of visual spatial cueing, including dynamic adjustment, and use during rehabilitation is recommended.

## Supporting information

S1 Fig **A scheme of the investigated path without (upper) and with 3 D visual cues (lower).** The marked investigated path was 10 m long with GaitRite Analysis System, a 4.9 m long pressure-sensitive walking mat, placed in the middle of the path. Sixteen squared wooden rods sized 2x2x100 cm were placed at distances of 60 cm across the walking pathway, perpendicular to the walking direction of the participants. PC; a computer connected to the GaitRite system, recording data from each gait trial.
(TIF)

S2 Fig (TIF)

S1 Table Characteristics of all recruited patients (n = 35). DBS, deep brain stimulation; L-Dopa Eq, levodopa equivalent; TEED, total electrical energy delivered; UPDRS, The Unified Parkinson's Disease Rating Scale; MMSE, Mini-Mental State Examination; FOGQ, Freezing of Gait Questionnaire; FES, Falls-Efficacy Scale. All recruited patients (n = 35).
(TIF)

S2 Table Temporal and special gait parameters of the PD patients and healthy controls. Values are mean ±SD (n = 25). Repeated measure ANCOVA factored for disease status corrected for leg length. Presented p values are post-hoc Tukey HSD test versus DBS OFF condition. #Controls versus patients were tested separately by one-factor ANCOVA with correction for leg length. * significance remained after Bonferroni correction for multiple testing DBS OFF, patients with deep brain stimulation turned off.
(TIF)
