# Supplementary figures and images for: 3D visual cueing shortens the double support phase of the gait cycle in patients with advanced Parkinson's disease treated with DBS of the STN
Source: PLoS One. 2020 Dec 31;15(12):e0244676. doi: 10.1371/journal.pone.0244676 (PMC7774936; doi:10.1371/journal.pone.0244676)

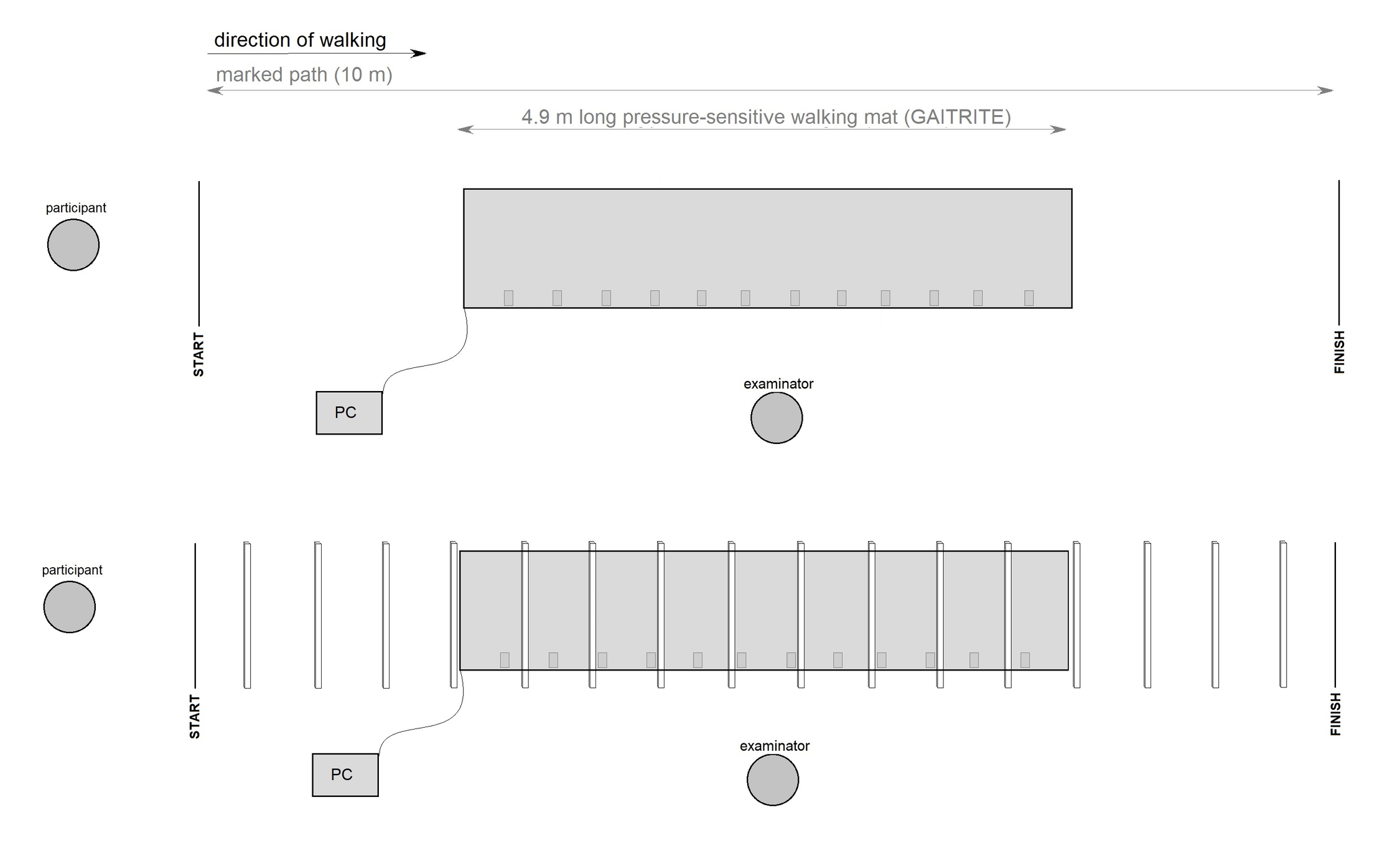

Supplement: S1 Fig — A scheme of the investigated path without (upper) and with 3 D visual cues (lower). The marked investigated path was 10 m long with GaitRite Analysis System, a 4.9 m long pressure-sensitive walking mat, placed in the middle of the path. Sixteen squared wooden rods sized 2x2x100 cm were placed at distances of 60 cm across the walking pathway, perpendicular to the walking direction of the participants. PC; a computer connected to the GaitRite system, recording data from each gait trial. (TIF) [file pone.0244676.s001.tif]

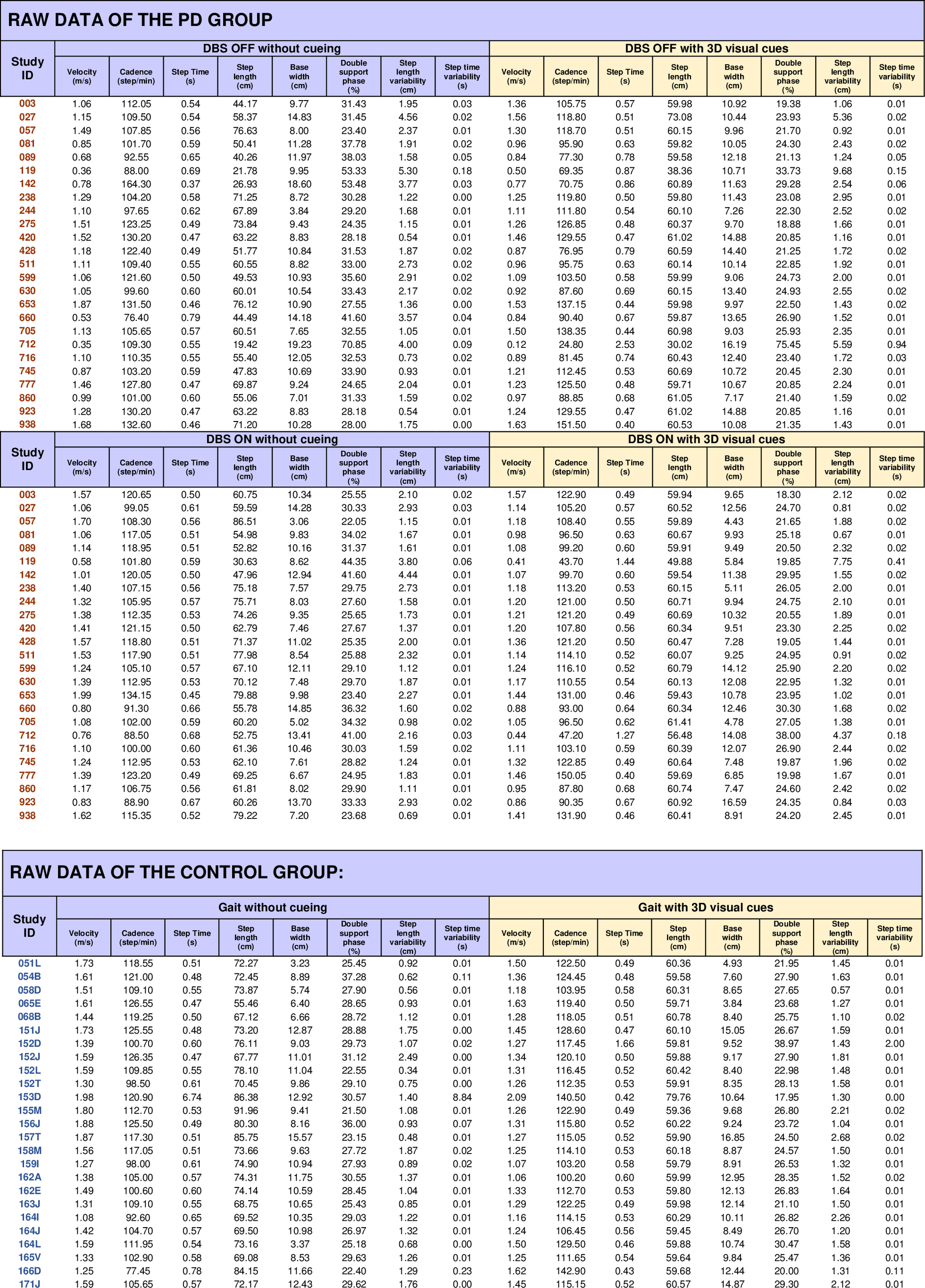

Supplement: S2 Fig — (TIF) [file pone.0244676.s002.tif]

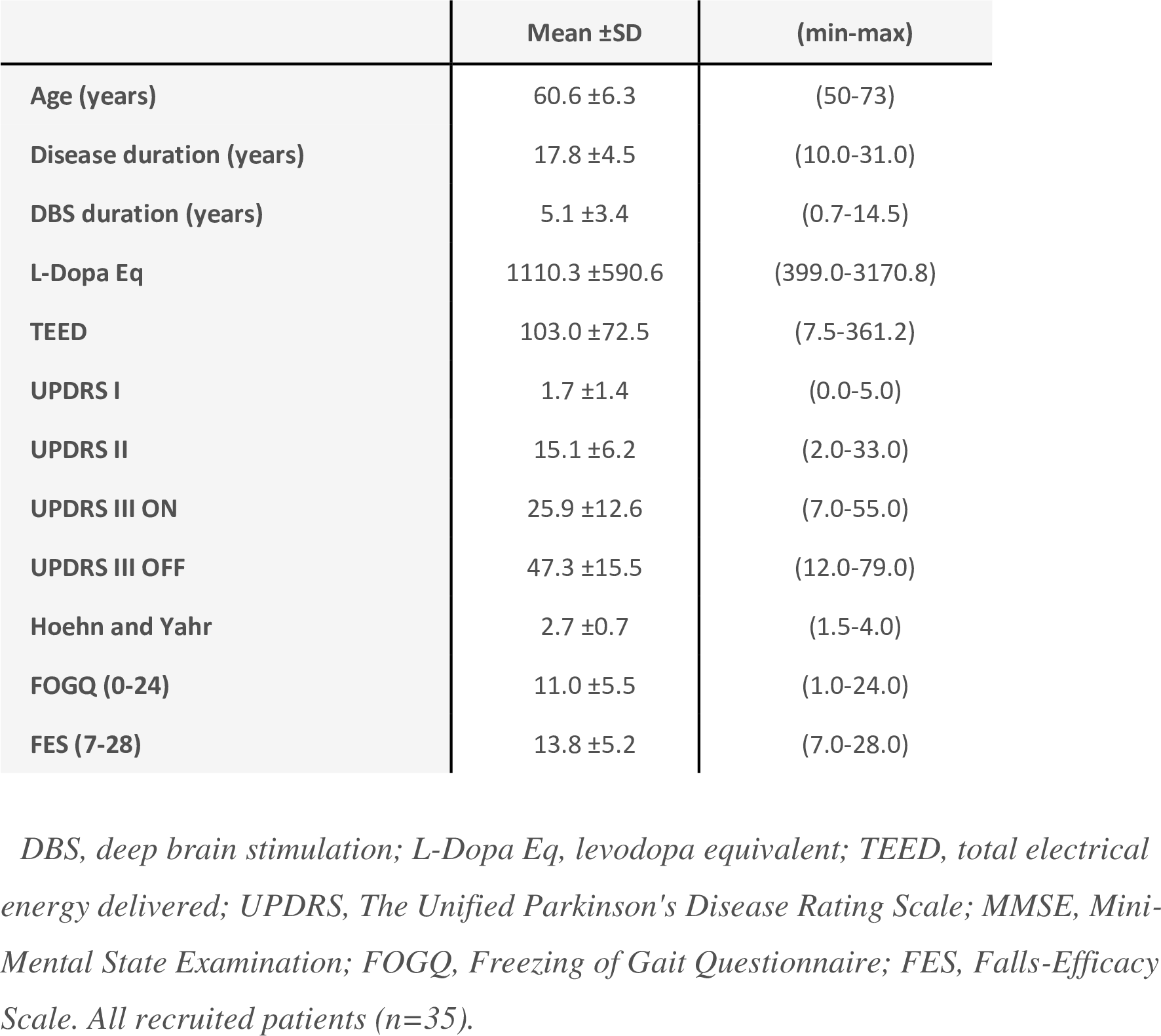

Supplement: S1 Table — DBS, deep brain stimulation; L-Dopa Eq, levodopa equivalent; TEED, total electrical energy delivered; UPDRS, The Unified Parkinson's Disease Rating Scale; MMSE, Mini-Mental State Examination; FOGQ, Freezing of Gait Questionnaire; FES, Falls-Efficacy Scale. All recruited patients (n = 35). (TIF) [file pone.0244676.s003.tif]

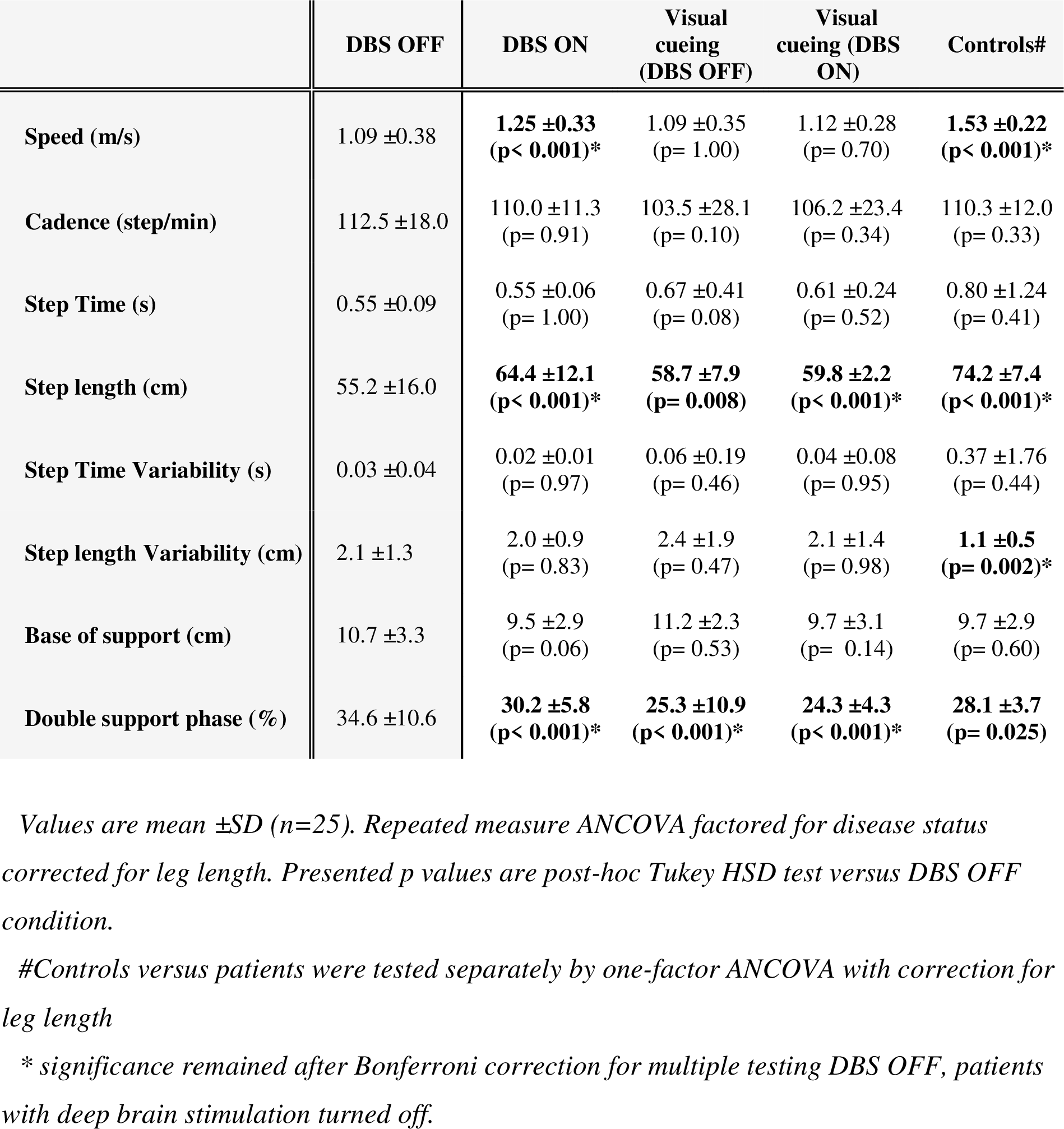

Supplement: S2 Table — Values are mean ±SD (n = 25). Repeated measure ANCOVA factored for disease status corrected for leg length. Presented p values are post-hoc Tukey HSD test versus DBS OFF condition. #Controls versus patients were tested separately by one-factor ANCOVA with correction for leg length. * significance remained after Bonferroni correction for multiple testing DBS OFF, patients with deep brain stimulation turned off. (TIF) [file pone.0244676.s004.tif]
